# A Peculiar Phospholipid-Binding Specificity and Cell Death-Related Properties of Tubic (*Nicotiana tabacum* Tubby-like F-Box Protein 8)

**DOI:** 10.3390/ijms27198474

**Published:** 2026-09-23

**Authors:** Grigoriy G. Safronov, Artemii A. Pigidanov, Raisa A. Galiullina, Irina E. Kovaleva, Anastasia V. Litvinova, Nina V. Chichkova, Andrey B. Vartapetian

**Affiliations:** 1 Belozersky Institute of Physico-Chemical Biology, Lomonosov Moscow State University, Moscow 119991, Russia; grigorij.safronov@gmail.com (G.G.S.); artemiy.pigidanov@mail.ru (A.A.P.); kovaleva@belozersky.msu.ru (I.E.K.); nasta.litwinova@gmail.com (A.V.L.); chic@belozersky.msu.ru (N.V.C.); 2 Faculty of Bioengineering and Bioinformatics, Lomonosov Moscow State University, Moscow 119991, Russia

**Keywords:** F-box domain, phospholipid-protein recognition, phytaspase, plant cell death, protein domain, plant stress, Tubby-like protein

## Abstract

Proteins possessing the Tubby domain (Tubby-like proteins, TLPs) are widespread in plants and represented by protein families. Although the function of TLPs is frequently associated with plant response to stresses, the mechanisms of their action are largely unknown. The recently demonstrated interaction of Tubic (*Nicotiana tabacum* Tubby-like F-box protein 8) with a cell death protease, phytaspase, prompted us to investigate the possible involvement of Tubic in plant cell death and to perform structural and functional characterization of distinct domains of Tubic. We demonstrated here a requirement for Tubic in the execution of the hypersensitive response of *Nicotiana benthamiana* plants induced by the INF1 protein of phytophthora. Also in line with the pro-death properties of Tubic was the observation that prolonged overexpression of Tubic compromised plant viability. Notably, inactivating mutations or complete deletion of the F-box domain markedly enhanced the cell death-inducing activity of Tubic. The N domain of Tubic turned out to be necessary for the observed down-regulation of phytaspase activity by Tubic overproduction. Cooperation between the N domain and the Tubby domain was apparently required to achieve maximum (approximately 2-fold) suppressive effect. In turn, for the Tubby domain, an expected capability to bind phospholipids was demonstrated, yet the specificity of this interaction was quite distinct from that reported for plant Tubby domains previously, with phosphatidic acid and phosphatidylinositol monophosphates being by far the strongest interactors. We propose that dissimilarity in phospholipid-binding specificity between the TLP family members may underlie the diversity in their responses to external cues.

## 1. Introduction

Tubby-like proteins are widely distributed across eukaryotes, including vertebrates, *Drosophila*, *Caenorhabditis elegans*, and plants [1]. A distinctive feature of these proteins is the presence of the so-called Tubby domain (~260 amino acid residues and possessing a characteristic fold) at their C-terminus [2,3]. As was first demonstrated for the mouse Tubby protein, it is the Tubby domain that enables Tubby to associate with the inner surface of the plasma membrane through recognition of membrane phosphoinositides, with a strong preference for binding to phosphatidylinositol 4,5-diphosphate (PtdIns(4,5)P2) [3]. Characteristic basic amino acid residues in the Tubby domain of Tubby were shown to be responsible for PtdIns(4,5)P2 recognition [3,4]. Membrane association of Tubby can thus be modulated by the availability of PtdIns(4,5)P2, a property that is regulated by phospholipases [5,6]. Activation of a G protein-coupled receptor leading to phospholipase C activation was demonstrated to trigger the release of Tubby and related Tubby-like proteins from the plasma membrane and their translocation into the nucleus [3].

The vertebrate family of Tubby-like proteins (TULPs) includes the founding member Tubby and related TULPs, TULP1 to TULP4 [1]. TULP1, a photoreceptor-specific protein, binds to phospholipids similarly to Tubby, although with less specificity [7]. Animal TULPs differ significantly in the nature of their N-terminal domains preceding the Tubby domain [8]. Proteins of this family are implicated in protein transport into cilia [8] and endocytic vesicle trafficking [9,10,11]. Mutations in mouse Tubby lead to maturity-onset obesity, blindness, and deafness [12,13].

In plants, Tubby-like proteins (commonly abbreviated as TLPs) are represented by larger gene families (e.g., 11 members in *Arabidopsis thaliana*, 14 in rice [14,15]). Tubby domains of at least some plant TLPs were reported to share the PtdIns(4,5)P2 binding capacity with their animal counterparts [16,17]. Furthermore, basic residues at the equivalent positions of plant and animal Tubby domains appear to mediate interaction with phospholipids [16,17].

In contrast to vertebrate TULPs, a common feature of TLPs is the almost ubiquitous presence of an F-box domain within these proteins, preceding the Tubby domain [14,18]. F-box proteins commonly function as substrate adaptors in SKP1-Cullin-F-box (SCF)-type E3 ubiquitin ligase complexes [19,20,21]. Therefore, plant TLPs may mediate the specific degradation of their partners. Indeed, *Arabidopsis thaliana* TLP6 was shown to target phosphoinositide biosynthesis-related kinases for ubiquitinylation and degradation [22]; tomato TLP8 promotes the ubiquitinylation and degradation of cyclin-dependent kinase B2 to orchestrate endoreduplication [23], while wheat TLP5 ubiquitinates catalase 1 resulting in reactive oxygen species (ROS) accumulation [24].

Available data suggest a role of plant TLPs in stress responses [16,22,24,25,26,27,28,29,30,31,32]. Overproduction of TLPs frequently enhances stress tolerance and positively regulates immune responses. TLPs have also been implicated in abscisic acid signaling [14,17]. However, the molecular mechanisms underlying the biological functions of TLPs, as well as their involvement in plant response to stresses, are largely unknown.

Recently, *N. tabacum* Tubby-like F-box protein 8 (named Tubic for brevity) was identified as a partner of a plant cell death-related protease, phytaspase [33]. This finding was supported by in vivo cross-linking experiments in *N. benthamiana* plants subjected to oxidative stress and by demonstrating direct in vitro interaction of phytaspase both with the Tubby domain of Tubic and with its N-terminal half (NF domain, Figure 1A). Phytaspase, a member of the plant subtilase family of proteases, participates in the accomplishment of programmed cell death induced by biotic and abiotic stresses [34,35,36,37] and possesses aspartate cleavage specificity and selectivity very similar to those of animal cell death proteases, caspases [38,39,40,41].

Like plant subtilases and unlike caspases, phytaspase is a constitutively active and extracellular protein under normal conditions [34,42]. However, upon the induction of death-promoting stresses, phytaspase utilizes clathrin-mediated endocytosis (CME) to re-enter plant cells to gain access to its intracellular targets [43,44]. Tubic was shown to be essential for this ‘retrograde transport’ of phytaspase, as suppression of Tubic levels by the VIGS prevented phytaspase internalization [33]. A mechanism allowing water-soluble extracellular phytaspase to employ CME for stress-induced retrograde transport is not well understood. Thus, identification of the plasma membrane-localized Tubic as a phytaspase interactor could potentially shed light on the mechanism of stress-induced internalization of phytaspase.

Also, as the interaction of Tubic with phytaspase appears to provide a link between Tubic functioning and the responses of plant cells to stress, here we strove to elucidate the roles and properties of Tubic domains that might be related to stress responses, particularly in relation to phytaspase. We demonstrated a peculiar phospholipid-binding specificity of Tubic (and of its Tubby domain) and a link between Tubic and the cell death process in plants mediated by all three domains of the protein.

## 2. Results

*N. tabacum* Tubic was recently discovered as a novel phytaspase interactor, both in vivo and in vitro [33]. To investigate the functional consequences that could arise from this interaction, we aimed to characterize Tubic properties and the roles of its specific domains (Tubby domain, F-box domain, and N domain, as in Figure 1A) in greater detail.

### 2.1. Tubby Domain of Tubic Displays Peculiar Phospholipid-Binding Properties

Because the Tubby domain of TLPs is known to confer specific binding of the proteins to the plasma membrane through the interaction with phospholipids, PtdIns(4,5)P2 in particular [3,16,17], we expected a similar behavior for Tubic. Our initial experiments seemed to support this assumption. The Tubic-EGFP protein produced in *N. benthamiana* leaf cells displayed peripheral localization and co-localized with a plasma membrane marker protein [45], LTI6b-mRFP—Figure 1B. Furthermore, fractionation of leaf extracts demonstrated that Tubic-EGFP is mainly present in the insoluble protein fraction, although a small amount of water-soluble Tubic-EGFP was also detected—Figure 1C (note that the LTI6b-mRFP protein was fully contained in the fraction of membrane proteins—Figure 1C, right panel).

To directly test the ability of Tubic to bind phospholipids, a recombinant protein MBP-Tubic (MBP, maltose-binding protein) was overproduced in *E. coli* cells, isolated by affinity chromatography on the amylose resin (Figure 1D), and used to assess its binding to an immobilized set of phospholipids in a PIP strip assay. Free MBP isolated in an identical fashion served as a negative control. This experiment confirmed the expected ability of Tubic to bind phospholipids (MBP-Tubic panel in Figure 1E). However, its specificity turned out to be substantially different from those reported for the mammalian tubby and for several TLPs of plant origin [3,17]. Instead of preferential binding to PtdIns(4,5)P2, Tubic demonstrated strong interaction with phosphatidic acid (PA) (but not with lysophosphatidic acid), somewhat weaker binding to PtdIns monophosphates, and much weaker binding to PtdIns di- or triphosphates (Figure 1E; see also Supplementary Figure S1 for semi-quantitative phospholipid-binding analysis using a PIP Array assay).

We hypothesized that the presence of the N- and F-box domains in Tubic could influence (alter) the phospholipid-binding specificity of the Tubby domain. To test this, a recombinant MBP-Tubby domain protein was constructed (Figure 1D) and used in a PIP strip assay. This analysis revealed that the strength and specificity of phospholipid-binding of full-length Tubic and of its Tubby domain were indistinguishable (compare MBP-Tubic and MBP-Tubby domain panels in Figure 1E).

We concluded that Tubic possesses an unusual phospholipid-binding specificity and that this specificity is fully determined by its Tubby domain.

### 2.2. A Search for an Amino Acid Motif in Tubic Involved in Phosphatidic Acid Interaction

For the canonical Tubby domain of the mammalian Tubby protein and for some plant TLPs, a prominent role of conserved Lys and Arg residues (K130 and R132, Tubby numbering) in phospholipid interaction has been reported [3,16,17]. These residues are conserved in Tubic as well (K188 and R190, Tubic numbering). To test their potential role in the Tubic–phospholipid interaction, we mutated both residues to alanine (the KR/AA mutant) and compared the phospholipid-binding capacity of the KR/AA mutant with that of the wild-type protein. As shown in Figure 2A, the mutations did not alter the strength or specificity of the Tubic interaction with phospholipids. Neither did they affect the peripheral localization of the protein in *N. benthamiana* leaf cells (Figure 2C), nor the presence of the protein in the membranous fraction upon extract fractionation (Figure 2D).

We then used several in silico docking algorithms to predict amino acid residues within the Tubby domain of Tubic likely participating in PA binding. An important validation criterion in this search was to confirm the experimentally observed specificity: efficient interaction with PA, no interaction with PtdIns, and restoration of binding to PtdInsP1 (see Figure 1E and Figure 2A). The most consistent prediction was that Arg130, Lys356, and Lys392 are likely to be involved in the interaction with an exposed phosphate group of the phospholipid (as shown in Figure 2B).

A triple Tubic mutant with substitution of the indicated basic residues with glutamate (the R2K/E mutant) was then constructed and experimentally tested for interaction with phospholipids. However, the PIP strip and PIP Array assays failed to reveal any change in the ability of the mutant protein to interact with phospholipids (Figure 2A, R2K/E panel; Supplementary Figure S1). Neither the intracellular localization of the mutant protein in *N. benthamiana* cells nor its presence in the membrane fraction (relative to the wild-type protein) was affected (Figure 2C,D), indicating that these Tubic residues are unlikely involved in phospholipid binding.

We concluded from these studies that the mode of Tubic–phospholipid interaction differs significantly, both in phospholipid specificity and in the amino acid motifs involved, from the behavior of the previously characterized TLPs.

### 2.3. Evaluation of the Impact of Tubic Domains in Accumulation of the Protein in Plant Cells

Tubic contains three domains that likely contribute to its function: the role of the Tubby domain was discussed above, with the preceding F-box domain implying a possible connection with the function of E3 ubiquitin ligases, and the N-terminal domain displaying no similarity to known protein domains (Figure 1A). To get an idea of the contribution of individual domains in Tubic function, we constructed a set of Tubic derivatives with one or two domains being deleted. These were: ΔN, ΔF, ΔNF (equivalent to the Tubby domain), ΔTubby (equivalent to the NF domain), and N domain (Figure 3A). Production of these Tubic derivatives in *N. benthamiana* leaf cells (as EGFP fusions) revealed that all Tubic mutants possessing the Tubby domain accumulated to relatively low levels and were markedly unstable, as inferred from their disappearance at 3 dpi (Figure 3B). Of note, deletion of the F-box domain (the ΔF mutant) failed to rescue the protein level within the cell.

On the other hand, elimination of the Tubby domain resulted in a profound accumulation of the ΔTubby protein (Figure 3B). Comparison of the behavior of the N domain with that of the NF domain (equivalent to ΔTubby) revealed that the N domain accumulated to an even higher level in plant cells, almost comparable to that of free EGFP (Figure 3C). Apart from this, the N domain appeared to be the only water-soluble portion of the Tubic protein (Figure 3C).

### 2.4. Properties of the Tubic F-Box Mutants: A Cell Death Connection

In *N. benthamiana* leaves producing Tubic-EGFP for a long period (4–5 dpi), cell death symptoms became visible (Figure 4A). Notably, the level of ectopically produced Tubic peaked at 2 dpi and decreased abruptly afterwards (Figure 3B and ref. [33]). These observations suggest that triggering the cell death process by Tubic occurred far in advance of its visible manifestation.

When the above-described Tubic mutants were individually produced in *N. benthamiana* leaves for a standard period of time (3 dpi), formation of necrotic lesions became visible for the ΔF mutant and also for the previously described LP/AA mutant with two amino acid substitutions likely affecting the F-box function [33]—Figure 4B. Leaf portions producing wild-type Tubic, ΔN, ΔNF, ΔTubby mutants, or free EGFP preserved viability. Notably, the KR/AA and R2K/E Tubic mutants described in Section 2.2 behaved more like wild-type Tubic, rather than like the ΔF and LP/AA Tubic mutants, in this assay (Supplementary Figure S2).

To further verify the initiation of cell death by the F-box mutants, ROS generation in *N. benthamiana* leaves producing wild-type Tubic, a full set of its derivatives, and free EGFP was assessed at 2 dpi by staining with DAB (diaminobenzidine). Both F-box mutants were observed to induce ROS production—Figure 4C. Consistently, staining of *N. benthamiana* leaves producing either Tubic-mRFP, Tubic LP/AA-mRFP, or free mRFP with H2DCFDA (2′,7′-dichlorodihydrofluorescein diacetate) revealed increased ROS production for Tubic-mRFP (relative to the free mRFP control), which was further enhanced for Tubic LP/AA-mRFP (Figure 4D; Supplementary Figure S3). Furthermore, production of the LP/AA mutant in *N. benthamiana* seedlings impaired cell viability, as became evident upon seedling staining with Evans Blue—Figure 4E. Therefore, inactivation of the F-box domain caused a strong increase in the pro-death activity of Tubic.

As the experiments with overproduced proteins may be prone to artifacts, we tested the involvement of Tubic in the cell death process in a different experimental system. INF1 protein of *Phytophthora infestans* is known to induce a hypersensitive response, a form of programmed cell death, in *N. benthamiana* [46,47]. We therefore compared INF1-induced cell death symptoms in Tubic-silenced plants (by using the VIGS approach, virus-induced gene silencing) versus control plants. To this end, recombinant INF1 protein was produced in *E. coli* cells and inoculated into *N. benthamiana* leaves of both types. Inoculation with INF1 caused characteristic necrotic lesions in the control leaves, as expected, whereas necrotization in the Tubic-silenced leaves was markedly suppressed—Figure 4F.

Collectively, these studies uncover a requirement for Tubic for the accomplishment of the hypersensitive response and a pro-death activity of overproduced Tubic. Furthermore, they demonstrated that the F-box mutants of Tubic possess an enhanced propensity to induce spontaneous death of plant cells.

### 2.5. Tubic Domains: A Phytaspase Connection

Our previous studies demonstrated the ability of the individual Tubby and NF domains of Tubic to interact with phytaspase in vitro (Figure 1A) and the capacity of the full-length Tubic to partially down-regulate phytaspase-specific proteolytic activity in vivo [33]. We now asked which structural elements in Tubic might be responsible for the in vivo attenuation of phytaspase activity.

We quantitatively determined the proteolytic activity of endogenous phytaspase in extracts obtained from *N. benthamiana* leaves producing either wild-type Tubic or the various above-mentioned derivatives thereof by using a phytaspase-specific fluorogenic peptide substrate, Ac-VEID-AFC [33]. The suppressive effect of wild-type Tubic was preserved in both F-box mutants, ΔF and LP/AA. However, elimination of the N-domain nullified the inhibitory effect, as exemplified by the behavior of ΔN and ΔNF derivatives—Figure 5. This finding supports a role for the N domain in the down-regulation of phytaspase activity.

On the other hand, the N domain or the NF domain (ΔTubby), although produced in plant cells to a very high level, exhibited either no inhibition (the N domain) or partial inhibition (the NF domain), but failed to reduce phytaspase activity to the same extent as full-length Tubic did—Figure 5. These data indicate that cooperation of the N domain with the Tubby domain is essential for the efficient phytaspase down-regulation.

## 3. Discussion

Identification of Tubic (*N. benthamiana* Tubby-like F-box protein 8) as a partner of the cell death-related protease, phytaspase, prompted a functional characterization of the individual Tubic domains. Our study uncovered several important issues.

Beginning with the N-terminal region of Tubic, the N domain was shown to be required for the observed down-regulation of phytaspase in *N. benthamiana* cells, as the deletion of this region abolishes the effect of Tubic overexpression on phytaspase. Interestingly, the N domain is necessary, but not sufficient, for suppressing phytaspase activity, and contribution from the Tubby domain is required for the full effect (Figure 5). These results are in agreement with previous in vitro data demonstrating that phytaspase can bind both to the N-terminal half of Tubic (NF domain) and to the Tubby domain in experiments with purified proteins. Notably, the N domain is highly stable in plant cells and is the only predominantly water-soluble portion of the Tubic protein.

The F-box domain separates the N and Tubby domains in Tubic. The presence of an F-box module in a protein is commonly regarded as an indication of its association with the SCF E3 ubiquitin ligase complex and involvement in the ubiquitin-dependent degradation of a protein partner of the F-box-containing protein. In addition, the presence of an F-box domain within the protein may also contribute to its own instability [48,49]. Although our previous studies demonstrated that Tubic is a fairly unstable protein [33], deletion of the F-box domain or mutating the residues likely involved in the interaction with E3 ubiquitin ligase did not substantially increase Tubic stability (Figure 3B and ref. [33]), nor did these mutations impair the ability of Tubic to down-regulate phytaspase activity in plant tissues (Figure 5). However, expression of ΔF and LP/AA mutants induced cell death in *N. benthamiana* leaves, resulting in enhanced ROS generation, plasma membrane damage, and formation of necrotic lesions (Figure 4).

Previous studies demonstrating a requirement for Tubic for oxidative stress-induced retrograde transport of phytaspase [33] linked Tubic to plant cell death. Our current results provide additional evidence by showing that overproduction of wild-type Tubic compromises the viability of plant cells during prolonged expression. This cell death-inducing capability is further enhanced for the F-box domain mutants. The conclusion that Tubic has pro-death properties is also supported by its requirement for the hypersensitive response in *N. benthamiana* triggered by *P. infestans* INF1 protein (Figure 4F). The scheme summarizing our current view on the possible role of Tubic in mediating plant cell death is presented in Figure 6. However, further studies are required to elucidate the molecular mechanisms underlying the role of Tubic in plant cell death and the reason why inactivation of its F-box domain converts Tubic into a potent inducer of plant cell death.

The Tubby domain exhibited several unexpected properties. Apart from the involvement of the Tubby domain, in cooperation with the N domain, in phytaspase activity down-regulation, the Tubby domain appears to determine the low abundance of Tubic in plant cells (Figure 3B). An exact reason for this behavior is not clear at present: this could be due either to inefficient translation of the region, or to the instability of the resulting protein.

The most striking feature of Tubic’s Tubby domain observed in the current study is its phospholipid-binding specificity. We confirmed here the expected capacity of Tubic (and of its Tubby domain) to bind phospholipids, but the specificity of this recognition turned out to be unforeseen and extends beyond phosphoinositides. Instead of preferential binding to PtdIns(4,5)P2, as reported for the prototype mouse Tubby and for some plant TLPs [3,17], the Tubby domain of Tubic demonstrated the strongest binding to phosphatidic acid (PA), somewhat lower binding to PtdIns monophosphates, and only weak binding to PtdIns di- or triphosphates (Figure 1E). PA, the simplest phospholipid, serves as a key metabolic intermediate and second messenger and plays a pivotal role in stress perception and downstream signal transduction under adverse conditions [50,51]. From a structural point of view, as no binding of Tubic to PtdIns could be detected, the presence of an exposed single phosphate group in the phospholipid appears to be crucial for this interaction. On the other hand, very strong binding to phosphatidic acid and complete lack of recognition of lysophosphatidic acid highlights the importance of a lipid component for Tubic binding as well.

In accord with the unusual specificity of phospholipid binding, the amino acid residues in Tubic involved in this interaction are also distinct from those reported previously for TLPs [3,16,17]. In particular, the KR/AA mutant completely preserved the ability and specificity of phospholipid interaction—Figure 2A. Identification of the Tubic residues involved is thus a reasonable goal for future studies.

Finally, TLPs in plants are encoded by multigene families, with approximately a dozen members for each plant species. The observed dissimilarity in phospholipid-binding specificity of various members of the family, demonstrated here for Tubic, suggests possible diversity of family members in the regulation of their association with cellular membranes and, consequently, in their biological functions, a possibility worth exploring.

## 4. Materials and Methods

### 4.1. Plant Growth Conditions and Agroinfiltration

*Nicotiana benthamiana* plants were grown at 25 °C in soil in a controlled environment under a 16 h/8 h day/night cycle. For transient protein production, *Agrobacterium tumefaciens* C58C1 cells were transformed with the respective plasmid. To enhance protein production in *N. benthamiana*, transformed cells were mixed with an equal number of agrobacteria bearing the p19 suppressor of silencing prior to infiltration. Agrobacteria carrying the empty vector were used as a control. Agrobacteria were infiltrated into the leaves of 6-week-old plants using a blunt syringe. Typically, leaves were harvested at 2 days post-infiltration (dpi), unless otherwise indicated in the Figure legends.

### 4.2. Plasmid Construction

Construction of the Tubic, Tubby domain, and LP/AA mutant genes and of the TRV2:NbTLP1 plasmid was described previously [33]. To obtain Tubic deletion mutants for in planta protein production, regions of Tubic cDNA were PCR-amplified using the following primer pairs: NtTubic_Nco_dir and Leader_Bam_rev (for the N domain); NtTubic_Nco_dir and Fbox_Bam_rev (for the NF domain); NtTub_deltaN_Nco_dir and NtTubic_Bam_rev (for ΔN); and NtTub_Tubdom_Nco_dir and NtTubic_Bam_rev (for ΔNF) (see Supplementary Table S1 for primer sequences). The DNA fragments thus obtained were used to substitute the Tubic gene in the pLEX_Tubic_EGFP expression plasmid [33]. For the ΔF Tubic mutant, the 5′-terminal DNA fragment of the Tubic ORF was amplified using primers pLH_seq_dir and Tub_leader_Afl_rev and inserted between the *Xho*I and *Afl*II sites of the pLEX_Tubic_EGFP plasmid. Schematic representations of the wild-type Tubic protein and of its deletion mutants constructed in this study (Figure 1A and Figure 3A) were created using the GNU Image Manipulation Program (GIMP), version 3.2.4 Community, Free Software (GPLv3), 2026 (https://www.gimp.org). The exact domain boundaries of the constructed Tubic derivatives are shown in Supplementary Table S2.

The KR/AA Tubic mutant cDNA was obtained via megaprimer PCR using an internal mutagenizing primer NtTubic_KR/AA_rev and the external NtTubic_Nco_dir primer (for the first PCR step) and NtTubic_Bam_rev primer (for the second step). To obtain the R2K/E Tubic mutant, mutations were sequentially introduced via megaprimer PCR using the mutagenizing primers Tubic_K356E_dir, Tubic_K392E_dir, and Tubic_R130E_Psi_rev. Subsequently, the wild-type Tubic gene in the pLEX-Tubic_EGFP and pET28a_MBP_Tubic plasmids [33] was substituted with the mutant genes.

The gene encoding the *P. infestans* INF1 protein was PCR-amplified using INF_Not_dir and INF_Sac_Xho_rev primers and total DNA from *Phytophthora*-infected tomato leaves as a template. The DNA fragment (approx. 300 bp long) was inserted downstream of and in frame with the His tag-encoding sequence of the pET28a bacterial expression vector (Novagen, Madison, WI, USA) to generate the pET28a_His_INF1 plasmid.

The identities of all constructs were confirmed using DNA sequence analysis (complete coding sequences were sequenced). Bacterial and in-plant expression of the constructed protein-encoding genes was driven by the T7 promoter and the dual cauliflower mosaic virus (CaMV) 35S promoter, respectively.

### 4.3. Transient Expression in Plants and Protein Fractionation

Leaves of *N. benthamiana* plants were infiltrated with agrobacteria carrying the constructed binary plasmids in combinations described in the Figure legends. At 2 dpi, leaf disks (approx. 15 mg each) from plants transiently producing the recombinant proteins were prepared and examined using confocal fluorescence microscopy.

For protein fractionation, the leaf material was frozen in liquid nitrogen and disrupted in a Minilys homogenizer (Bertin Instruments, Montigny-le-Bretonneux, France) using 1.4 mm zirconium oxide beads by five 5 s bursts. An additional 10 s burst was performed after suspending the samples in ICL buffer (10 mM Tris-HCl, pH 8.8, 0.2 M NaCl, 30 mM magnesium chloride, 0.2 M sucrose, and 10 mM 2-mercaptoethanol). Water-insoluble material was pelleted by centrifugation for 10 min at 14,000 *g* at 4 °C, and the supernatant obtained represented the water-soluble fraction of the intracellular proteins.

The pellets thus formed (the water-insoluble protein fraction) were solubilized in Sample Buffer and boiled for 10 min prior to analysis by PAGE. To obtain ‘total protein’ samples, leaf pieces were disrupted in a homogenizer as described above and, after the addition of Sample Buffer, boiled for 10 min. Supernatants obtained after centrifugation for 10 min at 14,000 *g* were applied to polyacrylamide gels.

### 4.4. Production of Recombinant Proteins in Escherichia coli Cells

*E. coli* BL21 (DE3) cells were transformed with pET28a-based constructs encoding either MBP-Tubic, MBP-Tubby domain, or free MBP and grown in 40 mL of LB medium containing 0.2% glucose and 25 μg/mL kanamycin at 37 °C with shaking. Exponentially growing cells were transferred to 18 °C, induced with 0.5 mM isopropyl-β-D-thiogalactoside, and further incubated overnight at 18 °C with shaking. Cells were pelleted by centrifugation at 4000 *g* for 15 min at 4 °C, resuspended in 5 mL of CB buffer (20 mM Tris-HCl, pH 7.4, 200 mM NaCl, 1 mM DTT, and 1 mM EDTA) containing the protease inhibitors aprotinin (5 μg/mL), chymostatin (40 μg/mL), and 4-(2-aminoethyl)benzenesulfonyl fluoride (25 µg/mL), sonicated on ice using five 20 s bursts, and centrifuged at 14,000 *g* for 10 min at 4 °C. The cleared lysate (1.5 mL) was incubated with Amylose resin (New England Biolabs, Ipswich, MA, USA) (100 μL of an aqueous suspension per experiment, pre-washed with CB buffer) for 1.5 h at 4 °C with rotation. The resin was then washed with 4 × 0.75 mL of CB buffer, and the bound proteins were eluted with 300 μL of CB buffer containing 10 mM maltose for 1 h at 4 °C.

For production of His-INF1, *E. coli* BL21 (DE3) cells transformed with the pET28a_His_INF1 plasmid were grown in 40 mL of Super medium containing 25 μg/mL kanamycin at 37 °C with shaking and induced with 0.5 mM isopropyl-β-D-thiogalactoside and harvested, as described above. The cell pellet was resuspended in 3 mL of extraction buffer (100 mM Tris HCl pH 8.8, 8 M urea, 100 mM NaCl, 0.1% Tween 20, and 5 mM β-mercaptoethanol). The suspension was heated for 4 min at 80 °C and centrifuged for 10 min at 14,000 *g* at room temperature. The supernatant was collected and mixed with 200 μL of Ni-NTA agarose (Qiagen, Hilden, Germany) equilibrated with extraction buffer. The mixture was incubated for 1 h at room temperature with shaking. The resin was then washed with 1 mL of wash buffer (100 mM Na-phosphate pH 6, 100 mM NaCl, 0.1% Tween 20, and 5 mM β-mercaptoethanol) 10 times with descending concentrations of urea. The resin was incubated in 150 μL of wash buffer with 100 mM EDTA at 4 °C for 30 min for the first elution and for 1 h for the second elution.

### 4.5. Phospholipid-Binding Assay

Phospholipid-binding assays were performed with commercially available kits (PIP strip and PIP Array; Echelon Biosciences, Salt Lake City, UT, USA) according to the manufacturer’s protocol. The membrane was blocked with TBST buffer (20 mM Tris-HCl, pH 7.5, 140 mM NaCl, and 0.1% Tween 20) containing 3% BSA for 1 h, incubated with the protein of interest (0.5 μg/mL) in 10 mL of TBST + 3% BSA buffer for 2 h at room temperature, then washed with 3 × 10 mL TBST + 3% BSA. The membrane was then consecutively incubated with mouse monoclonal anti-MBP antibody (New England Biolabs, Ipswich, MA, USA, at 1:10,000 dilution) and HRP-labeled anti-mouse secondary antibodies (Thermo Scientific, Waltham, MA, USA, at 1:10,000 dilution) before detection with ECL reagent, as described in Section 4.10.

### 4.6. Visualization of Cell Death Symptoms

For ROS detection, a *N. benthamiana* leaf producing Tubic derivatives (at 2 dpi) was vacuum infiltrated with 1 mg/mL DAB (3,3′-diaminobenzidine; Sigma, St. Louis, MO, USA) in 10 mM Na_2_HPO_4_ and incubated in DAB solution with gentle agitation for 12 h in the dark. The leaf was then bleached by incubating in 96% ethanol for 20 min with heating. Ethanol was removed by washing with 70, 50, and 20% ethanol and water, successively. Photographs were taken with bottom illumination.

Alternatively, leaf pieces were infiltrated with 2 μM aqueous H2DCFDA solution (2′,7′-dichlorodihydrofluorescein diacetate; Lumiprobe, Moscow, Russia), incubated in the solution with gentle agitation for 2 h in the dark, and inspected using confocal fluorescence microscopy (excitation wavelength: 488 nm).

To assess plasma membrane integrity, seven-day-old sterile *N. benthamiana* seedlings were vacuum infiltrated with transformed agrobacteria, and three days after infiltration the seedlings were stained with Evans Blue (Sigma, St. Louis, MO, USA) according to [44]. Briefly, detached seedlings were vacuum-infiltrated with a fresh aqueous solution of 0.5% Evans Blue and incubated for 15 min at room temperature, then washed three times for 15 min with water, with gentle shaking to remove unbound dye. Chlorophyll was removed by incubating samples in 96% ethanol for 10 min at room temperature twice, followed by washing with 70, 50, and 20% ethanol and water, successively.

INF1 preparation was diluted to a final concentration of 1 μg/mL (100 nM) in buffer containing 10 mM MES pH 5.5, 100 mM NaCl, 10 mM MgCl_2_, and 0.01% Silwet L-77. The solution was infiltrated into *N. benthamiana* leaves using a syringe without a needle. After necrosis appeared on the leaves, lesions were photographed under UV light (365 nm) using a UVP BioSpectrum Imaging System (UVP, Upland, CA, USA). The areas of infiltration and necrosis were measured using ImageJ/Fijiv.1.54.

### 4.7. Confocal Fluorescence Microscopy

Microscopic imaging was performed using a Zeiss LSM900 Airyscan (Carl Zeiss, Oberkochen, Germany) confocal microscope equipped with 488 and 590 nm lasers for excitation of EGFP and mRFP, respectively. Images were acquired using a Plan-Apochromat 20×/0.8 lens (Carl Zeiss, Oberkochen, Germany). Identical microscope settings were maintained across all experimental groups. Data were reproducible over at least three independent experiments. Confocal images were processed using ZEN Blue v. 3.4.91 (Carl Zeiss, Oberkochen, Germany).

### 4.8. Phytaspase Activity Determination

The proteolytic activity of phytaspase in total *N. benthamiana* leaf extracts was determined using the Ac-VEID-AFC [AFC, 7-amino-4-(trifluoromethyl) coumarin] fluorogenic peptide substrate (California Peptide Research, Salt Lake City, UT, USA), as described in [52]. The peptide substrate was used at a final concentration of 20 μM. Protein samples were diluted twofold before activity measurements. Kinetic measurements of relative fluorescence increase were performed in B1 buffer, pH 5.5, containing 0.5 M NaCl at 30 °C. The FLUOstar OPTIMA reader (BMG Labtech, Ortenberg, Germany) equipped with 405 nm excitation and 520 nm emission filters was used to quantify fluorescence intensities. Data are presented as means ± SD from three independent experiments. Statistical significance was analyzed using a *t*-test with Benjamini–Hochberg correction. *p* < 0.05 was considered significant.

### 4.9. Virus-Induced Gene Silencing

Generation of Tubic-silenced *N. benthamiana* plants was performed as described previously [33]. Briefly, Tobacco Rattle Virus (TRV) derivatives targeting two distinct regions of *N. benthamiana* Tubic mRNA were employed. *Agrobacterium* C58C1 cultures containing TRV1 [53,54] and TRV2:NbTLP1 or TRV2:NbTLP1&14 [33] were mixed at a 1:1 ratio and infiltrated into *N. benthamiana* leaves at the four-leaf stage. TRV1 with an empty TRV2 vector (TRV:00) served as the control. Approximately one month after viral inoculation, total RNA samples were obtained and used to determine the relative levels of NbTLP1 mRNA by real-time PCR with NbTub1_qPCR_dir, NbTub14_qPCR_dir, and NbTub_qPCR_rev primers. Typically, a two-to-five-fold reduction in the Tubic mRNA level was achieved with both constructs [33], and similar functional results were obtained upon INF1 infiltration (100 nM) in the silenced leaves.

### 4.10. Western Blot Analysis

After the addition of 5xSample Buffer (250 mM Tris-HCl, pH 6.8, 5 mM EDTA, 10% SDS, 30% glycerol, and 50 mM DTT), samples were boiled for 10 min and fractionated by SDS-polyacrylamide gel electrophoresis (10% gels were used) [55]. Separated proteins were electrophoretically transferred onto a polyvinylidene fluoride (PVDF) membrane. Detection of the EGFP- and mRFP-containing proteins was performed using monoclonal anti-EGFP 3A9 antibody [56] and rabbit polyclonal anti-tRFP antibodies (Evrogen, Moscow, Russia; at 1:2000 dilution), respectively, in combination with secondary anti-mouse or anti-rabbit IgG linked to HRP (Thermo Scientific, Waltham, MA, USA). Chemiluminescence detection was performed with Clarity Western ECL Substrate using the ChemiDoc Imaging System (Bio-Rad Laboratories, Hercules, CA, USA).

### 4.11. Computational Modeling of the Protein Structure and Protein–Ligand Complex

The three-dimensional structure of the Tubby domain was predicted using AlphaFold 3 [57]. The resulting protein structure was used as the starting model for subsequent modeling of its interaction with phosphatidic acid (PA). Protein–ligand complex modeling was performed using several independent computational approaches, including Boltz-2 [58], Chai-1 [59], IntelliFold [60], Protenix [61], and DynamicBind [62]. In all cases, the amino acid sequence of the protein and the three-dimensional protein structure predicted using AlphaFold 3 were used as input data. Each resulting three-dimensional complex model was independently analyzed to characterize the interaction between the protein and phosphatidic acid. Amino acid residues located within 4 Å of any ligand atom were considered potential ligand-interacting residues. For each model, the composition of these residues was determined, followed by a comparative analysis of their occurrence across models generated using the different computational approaches. The analysis revealed a high degree of consistency between the predictions, with the same amino acid residues being among those most frequently identified in close proximity to the ligand across the models generated by the different methods (Supplementary Tables S3 and S4). Based on the consistency of the results obtained using the different modeling approaches, one of the protein–ligand complex models generated using Chai-1 was selected for visualization. Three-dimensional visualization of the complex and analysis of the spatial arrangement of amino acid residues relative to the ligand were performed using PyMOL (version 4.6.0, Schrödinger, LLC.).

## Figures and Tables

**Figure 1:**
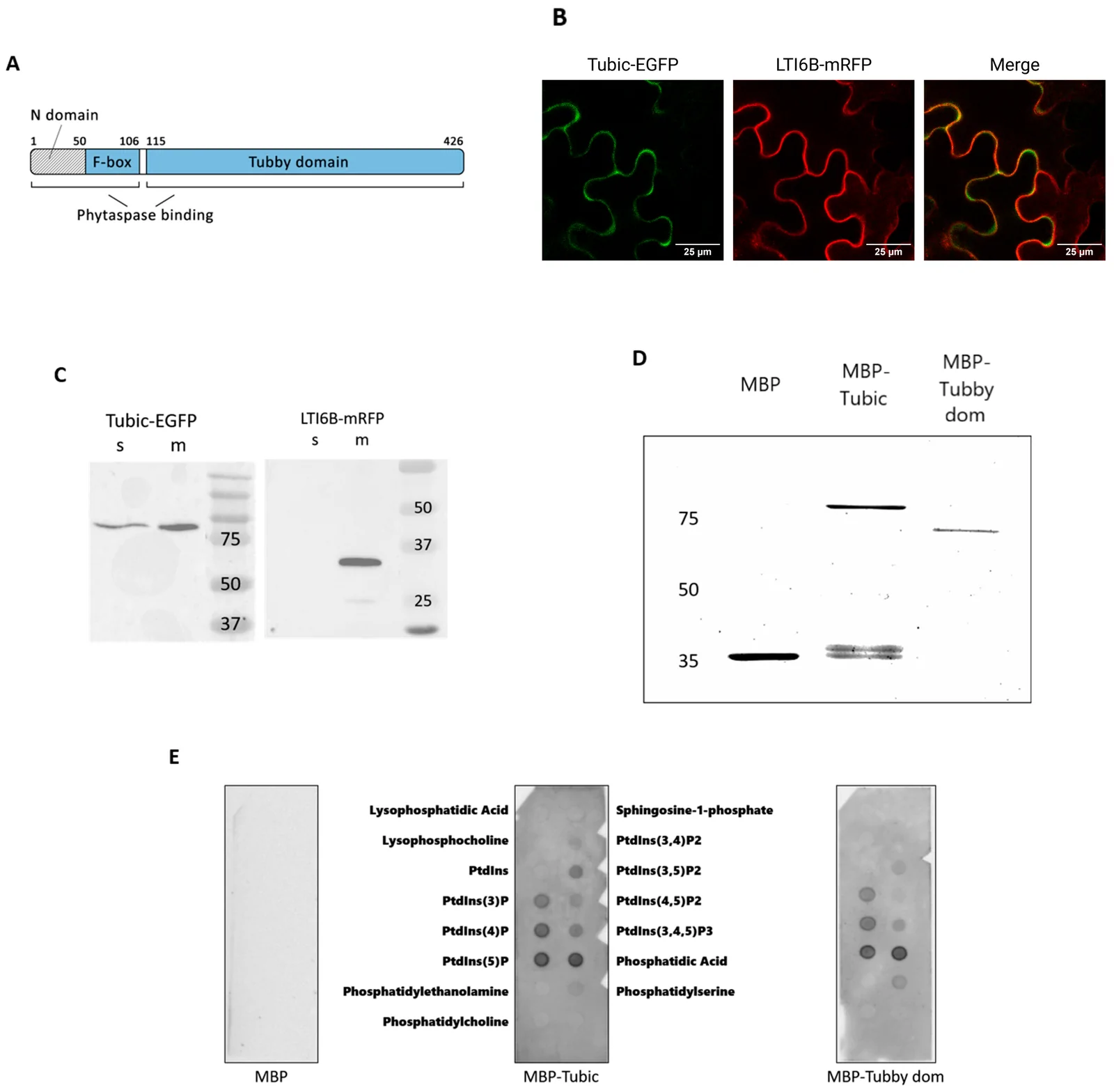
Tubic: Evidence for membrane localization and phospholipid binding. (**A**). Domain structure of Tubic. Tubic regions shown to bind phytaspase in vitro are indicated. (**B**). Confocal microscopy of Tubic-EGFP (green fluorescence, left panel) and LTI6b-mRFP (red fluorescence, middle panel) co-produced in *N. benthamiana* leaf cells by agroinfiltration at 2 dpi. (**C**). Separation of protein extracts from the leaves shown in (**B**) into water-soluble (s) and water-insoluble (presumably membrane (m)) protein fractions. Western blot (WB) analysis was performed using anti-EGFP (left panel) and anti-mRFP (right panel) antibodies. (**D**). Samples of MBP, MBP-Tubic, and MBP-Tubby domain proteins used for PIP strip binding assays. Proteins were separated by SDS-PAGE on a 10% polyacrylamide gel and visualized by Coomassie blue staining. (**E**). Phospholipid-binding assays for MBP-Tubic, MBP-Tubby domain, and free MBP. Following incubation of PIP strip membranes with equal amounts of the test protein, bound proteins were visualized using anti-MBP antibody and chemiluminescence detection. The positions of 15 individual phospholipids in each strip are indicated in the central image. Note that no detectable background binding to the membrane was observed with the free MBP probe (left image).

**Figure 2:**
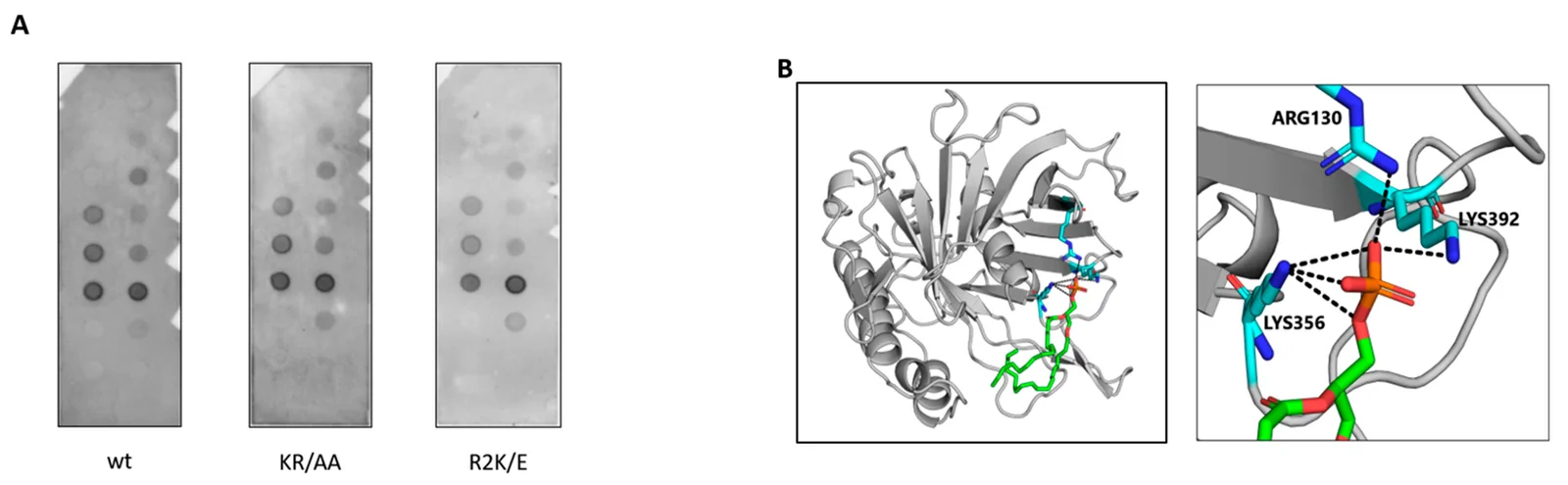

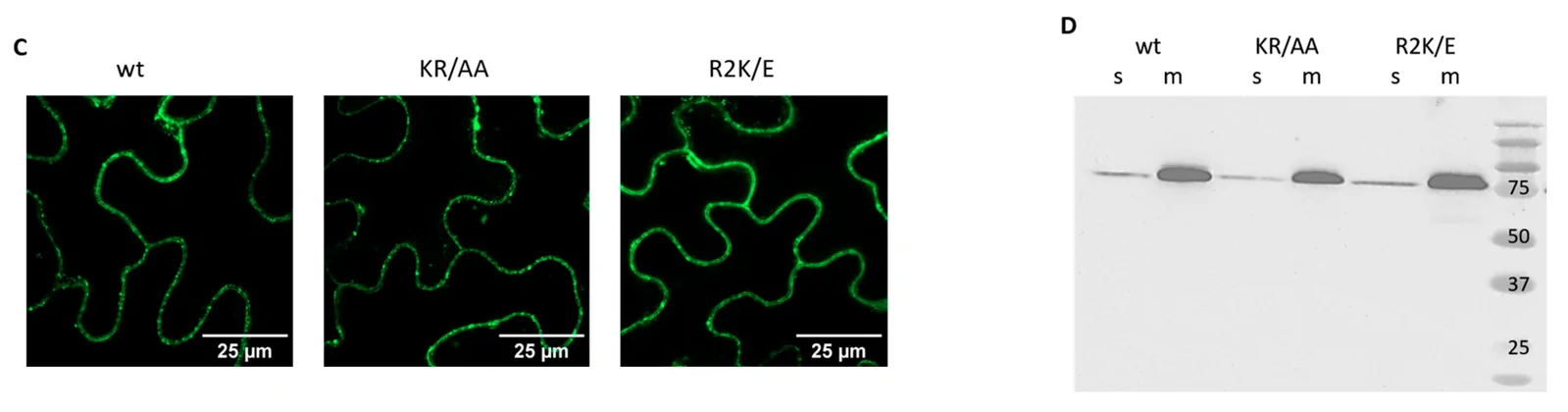
A search for basic residues within the Tubby domain of Tubic involved in phospholipid interaction. (**A**). PIP strip binding assays for wild-type Tubic (wt, the same image as the central image for MBP-Tubic in Figure 1E) and its point mutants KR/AA and R2K/E. (**B**). Computer modeling of the interaction of phosphatidic acid (PA) with the Tubby domain of Tubic. The Tubby domain is colored light gray; PA is colored green and red. The right panel shows the positions of the basic residues (colored blue) predicted by several algorithms to interact with the phosphate group of PA. (**C**). Confocal fluorescence microscopy of Tubic-EGFP (wt), Tubic KR/AA-EGFP, and Tubic R2K/E in *N. benthamiana* leaf cells at 2 dpi. (**D**). Membrane binding by Tubic is not affected by the KR/AA and R2K/E mutations. The soluble protein (s) and water-insoluble protein (m) fractions from leaf pieces producing the respective Tubic derivative were separated by 10% SDS PAGE. WB visualization was performed using an anti-EGFP antibody.

**Figure 3:**
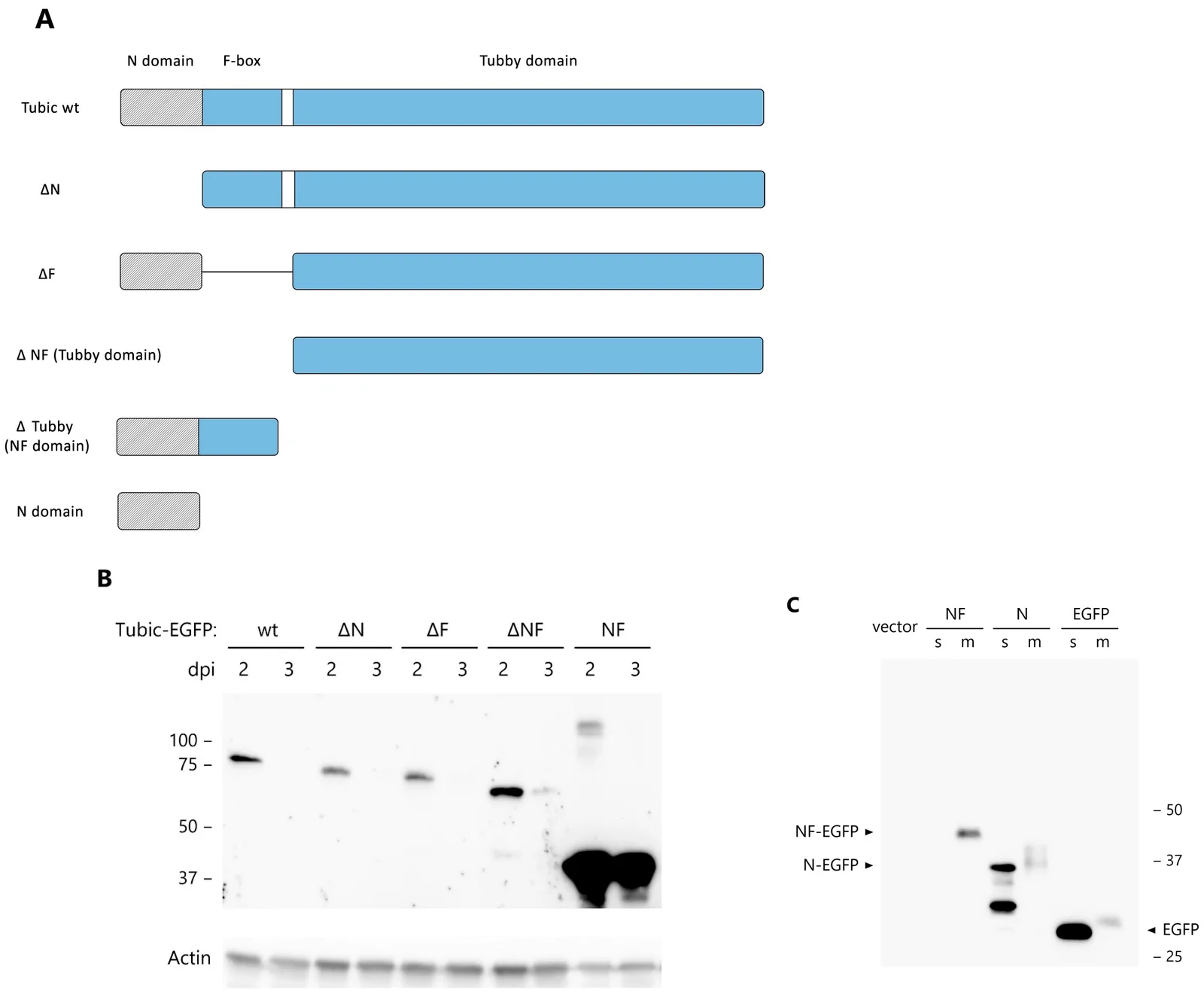
Properties of Tubic deletion mutants. (**A**). Schematic representation of the Tubic deletion mutants constructed in this study. (**B**). Relative accumulation levels of Tubic-EGFP (wt) and its deletion mutants produced in *N. benthamiana* leaves by agroinfiltration at 2 and 3 dpi. Leaf extracts were separated by 10% SDS-PAGE. WB visualization was carried out with an anti-EGFP antibody. Bottom panel: loading control with anti-actin antibodies. (**C**). Comparison of the membrane-binding properties of the N and NF domains of Tubic. The soluble (s) and water-insoluble (presumably membrane (m)) protein fractions from leaves producing each Tubic derivative as an EGFP fusion, or free EGFP as a control, were obtained at 2 dpi and separated and analyzed as in (**B**).

**Figure 4:**
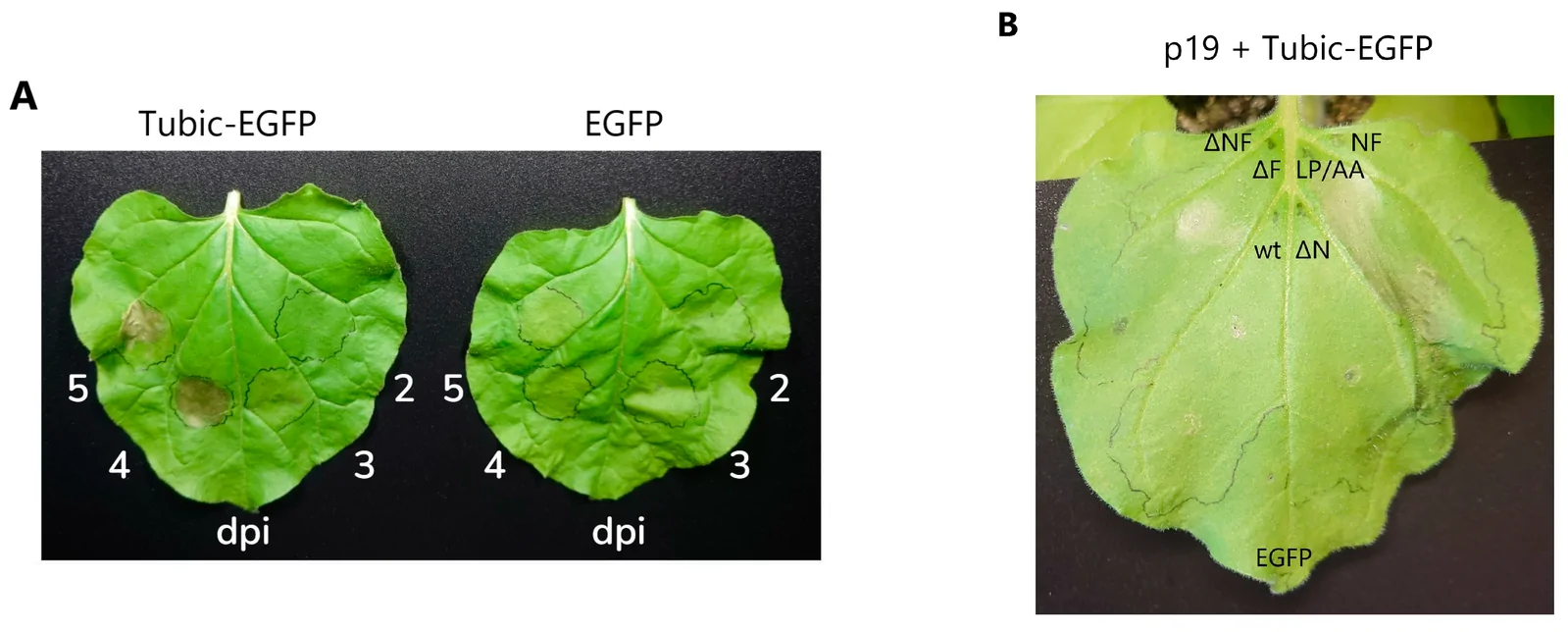

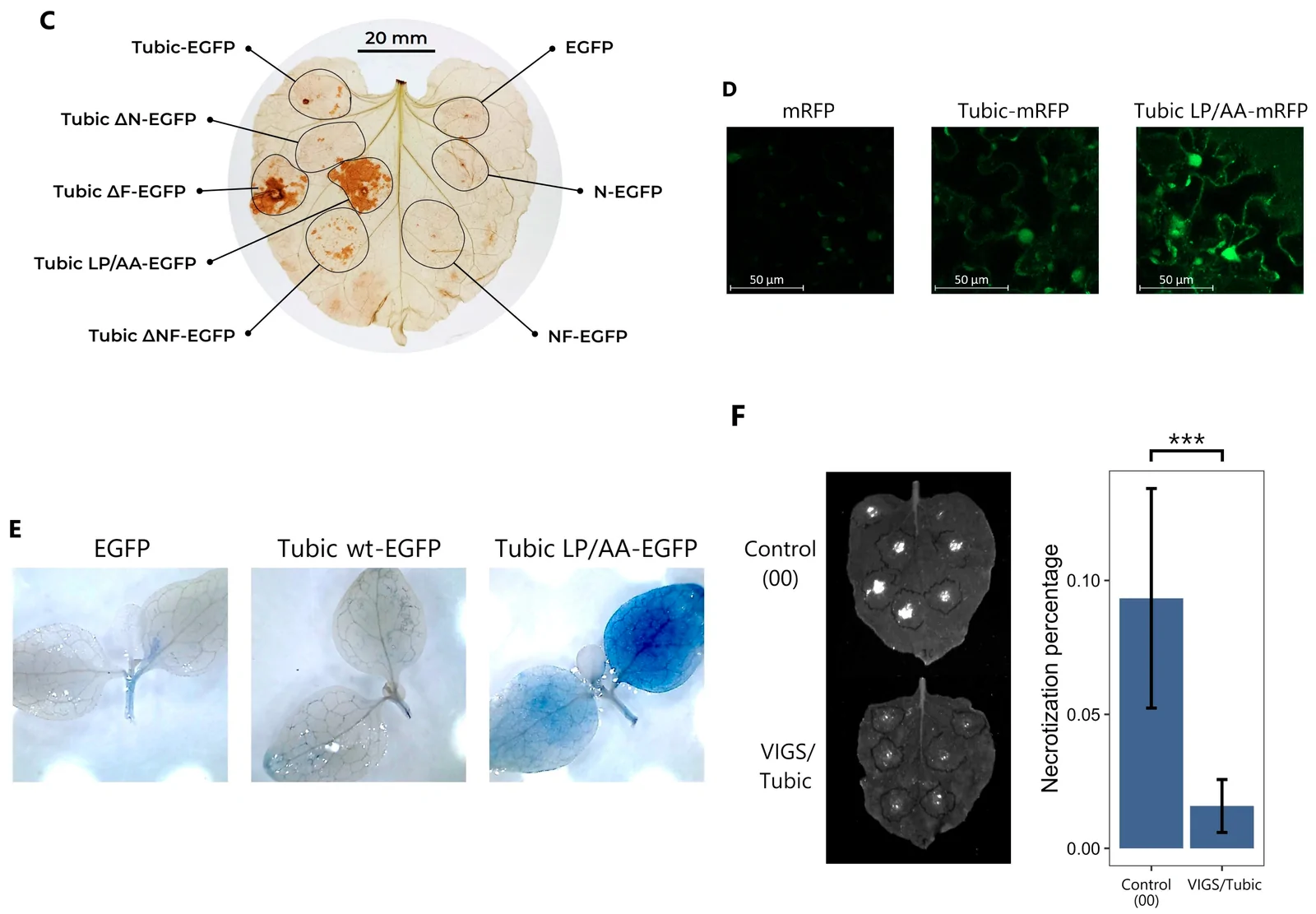
Tubic: A cell death connection. (**A**). Transient overproduction of Tubic-EGFP compromises the viability of *N. benthamiana* leaf cells at late time points after infiltration. (**B**). Production of the F-box Tubic mutants (EGFP fusion proteins), ΔF and LP/AA, causes early necrotization in an *N. benthamiana* leaf, whereas leaf areas producing wild-type (wt), ΔN, ΔNF, and ΔTubby domain proteins preserve cell viability. An image was obtained at 3 dpi. (**C**). Detection of ROS in *N. benthamiana* leaf areas producing Tubic derivatives as EGFP fusions at 2 dpi using diaminobenzidine staining. (**D**). Fluorescence microscopy detection of ROS in *N. benthamiana* leaves producing Tubic-mRFP, Tubic LP/AA-mRFP, or free mRFP at 2 dpi using H2DCFDA staining. (**E**). Evans blue staining of 7-day-old *N. benthamiana* seedlings producing either EGFP, Tubic wt-EGFP, or Tubic LP/AA-EGFP for 2 days. (**F**). Formation of necrotic lesions in *N. benthamiana* leaves induced by infiltration with INF1 protein (100 nM) is suppressed in Tubic-silenced (by VIGS) plants, relative to the control plants infected with the empty virus vector, 00. The right panel shows quantification of the necrotic areas. Data are presented as means ± SD from biological replicates; statistical significance was analyzed using a *t*-test, (***) represents *p* < 0.001. All the experiments shown in this Figure (panels (**A**–**F**)) were independently repeated at least 3 times with similar results.

**Figure 5:**
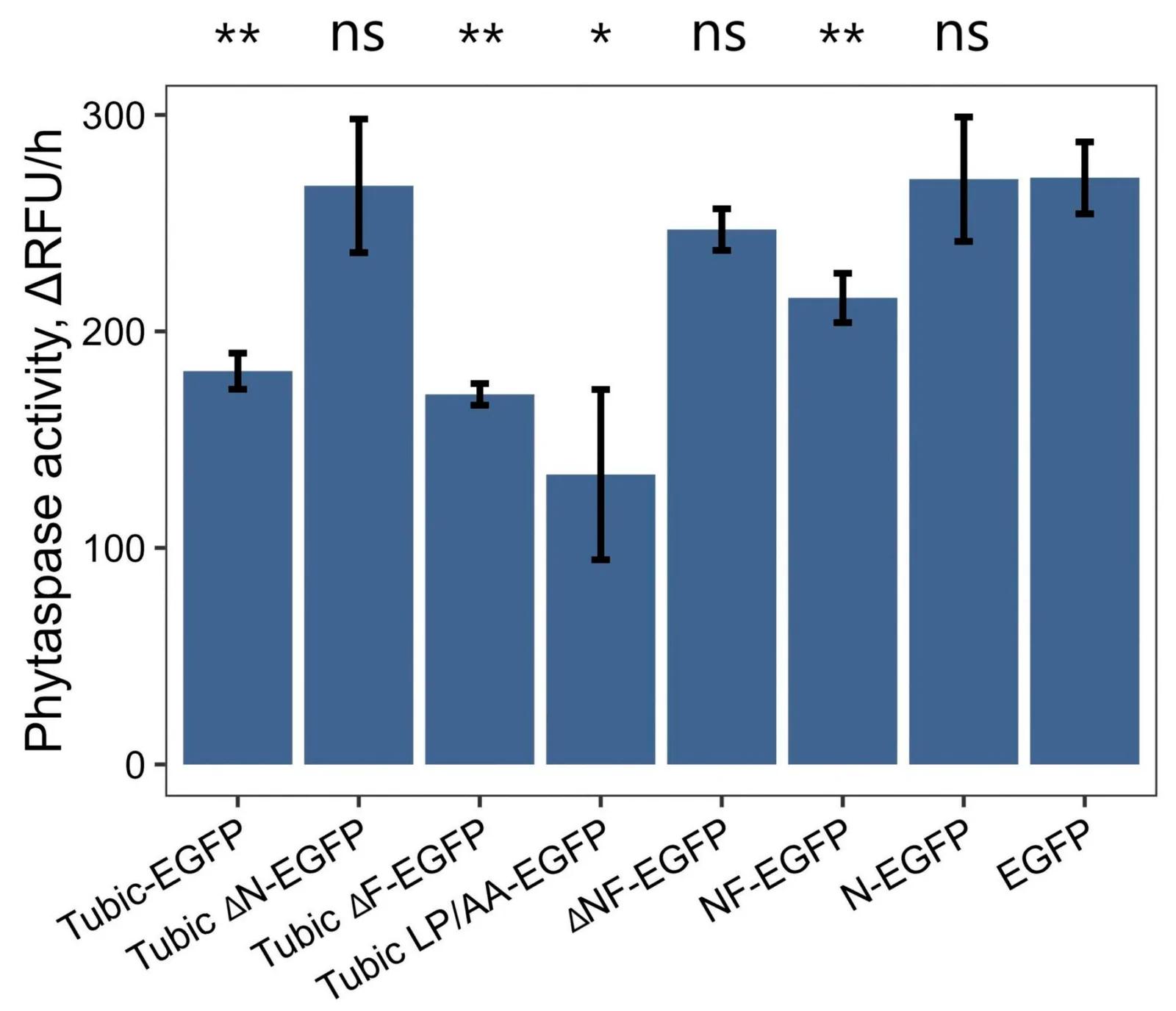
Differential down-regulation of phytaspase proteolytic activity by Tubic mutants. Leaf extracts were obtained from *N. benthamiana* leaves producing Tubic-EGFP and its mutants at 2 dpi and analyzed for phytaspase-specific proteolytic activity using 20 µM Ac-VEID-AFC as a fluorogenic peptide substrate. Relative rates of hydrolysis were determined as increases in relative fluorescence units per hour (ΔRFU/h). Data represent the mean ± SD from three independent experiments. In each experiment, 3–4 biological replicates were analyzed, and the data were reported as median values. Each of the 7 test groups was compared with the control group (EGFP production). Statistical significance was assessed using a *t*-test with Benjamini–Hochberg correction. (*) represents *p* < 0.05; (**) represents *p* < 0.01; ns, not significant.

**Figure 6:**
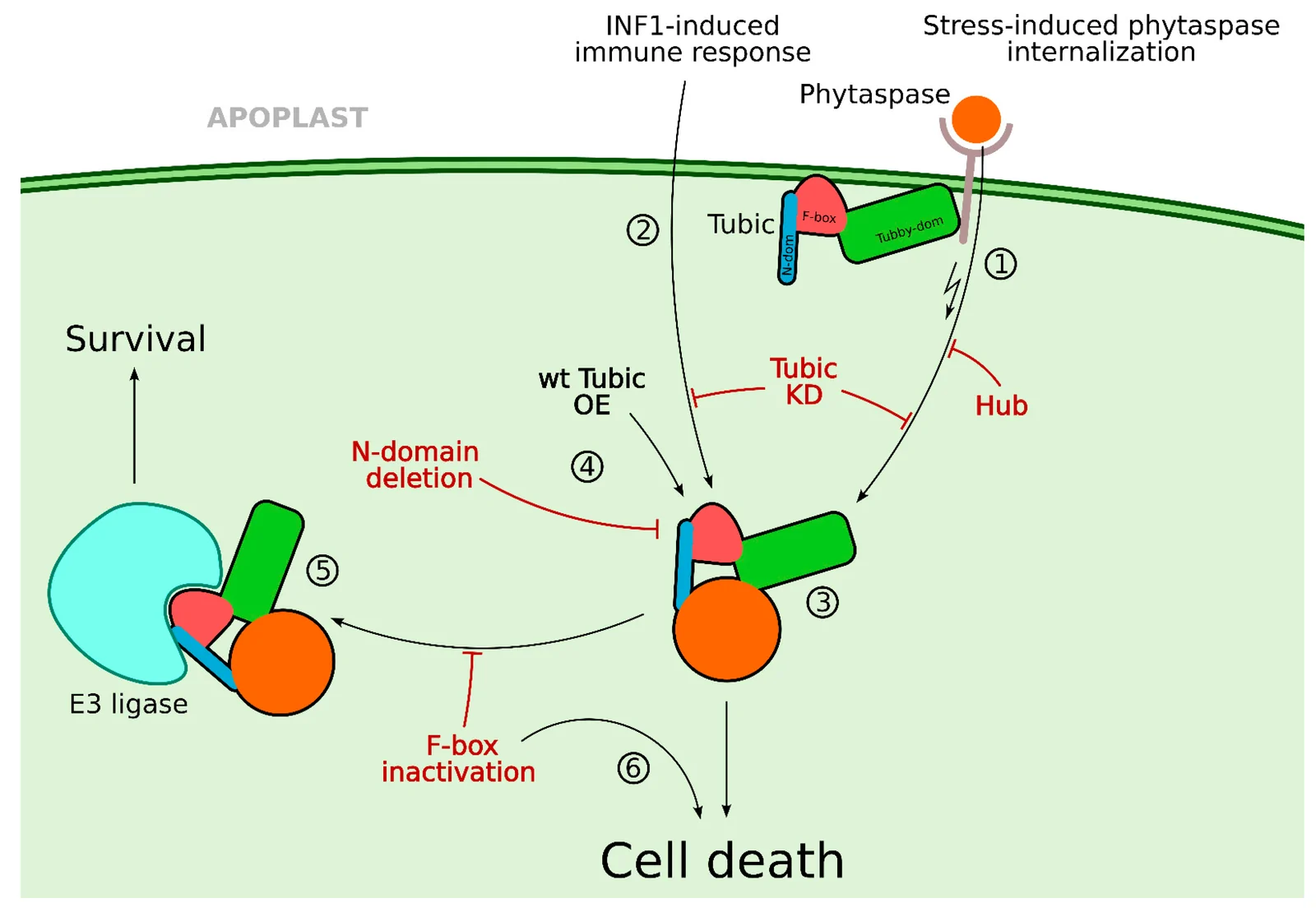
Tubic involvement in plant cell death: facts and hypotheses. Based on the current and previous [33,34,43,44] studies, we propose that the involvement of Tubic in the regulation of plant cell death could be multifaceted. (1) Being an intracellular and plasma membrane-localized protein, Tubic is required for clathrin-mediated endocytosis (CME) of water-soluble apoplastic phytaspase (likely bound to its hypothetical receptor) in response to stress-inducing stimuli, possibly acting as an adaptor protein. Knockdown (KD) of Tubic or suppression of the CME by the Hub protein prevents phytaspase internalization and cell death. (2) Likewise, knockdown of Tubic suppresses cell death induced by the INF1 protein of *Phytophthora*, thus implying a role for Tubic in the plant immune response. (3) The interaction of Tubic with phytaspase was documented both in vivo and in vitro, and the N and Tubby domains of Tubic appear to be involved in this interaction. We propose that a complex formed by Tubic with internalized phytaspase may be an active player in triggering cell death. (4) Such an assumption would explain why overexpression (OE) of wild-type Tubic (but not of its ΔN mutant) compromises viability of plant cells. This death-promoting effect is, however, moderate due to the low availability of intracellular phytaspase in non-stressed cells and/or to the down-regulation of its level by Tubic. (5) The presence of an F-box domain in Tubic hints at a possible interaction between the Tubic-phytaspase complex and an E3 ubiquitin ligase, followed by degradation of one or both of its constituents, thus fostering cell survival. (6) Inactivation of Tubic’s F-box stabilizes the complex, thus leading to accelerated cell death. The proposed scheme looks excessively speculative at the moment, although it allows us to make testable predictions that can be addressed in future studies.

## Data Availability

The original contributions presented in this study are included in the article/Supplementary Material. Further inquiries can be directed to the corresponding author.

## Supplementary Materials

The following supporting information can be downloaded at: https://www.mdpi.com/article/10.3390/ijms27198474/s1.
