# Supplementary material for: A Peculiar Phospholipid-Binding Specificity and Cell Death-Related Properties of Tubic (Nicotiana tabacum Tubby-like F-Box Protein 8)
Source: Int J Mol Sci. 2026 Sep 23;27(19):8474. doi: 10.3390/ijms27198474 (PMC13665275; doi:10.3390/ijms27198474)
Supplement: Supplementary file 1 [file ijms-27-08474-s001.zip › ijms-4552678-supplementary.pdf]

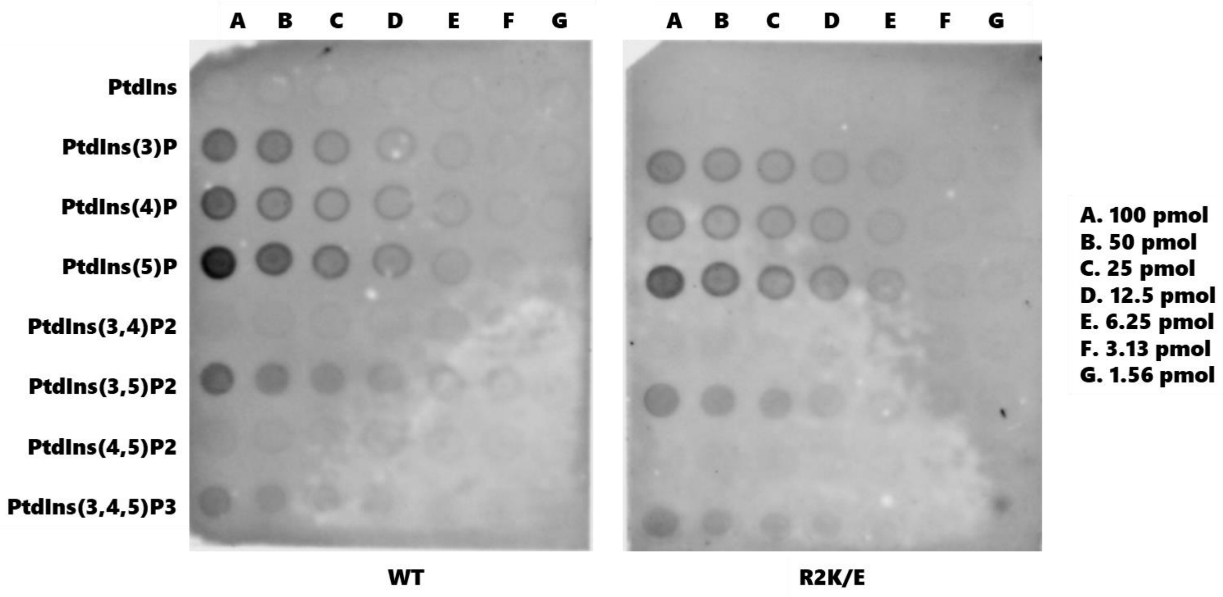

Supplementary Figure S1. A PIP Array assay of phospholipid binding for wild type Tubic (wt) and its R2K/E mutant (R2K/E). Amounts of phospholipids in each column (A to G) are indicated on the right.

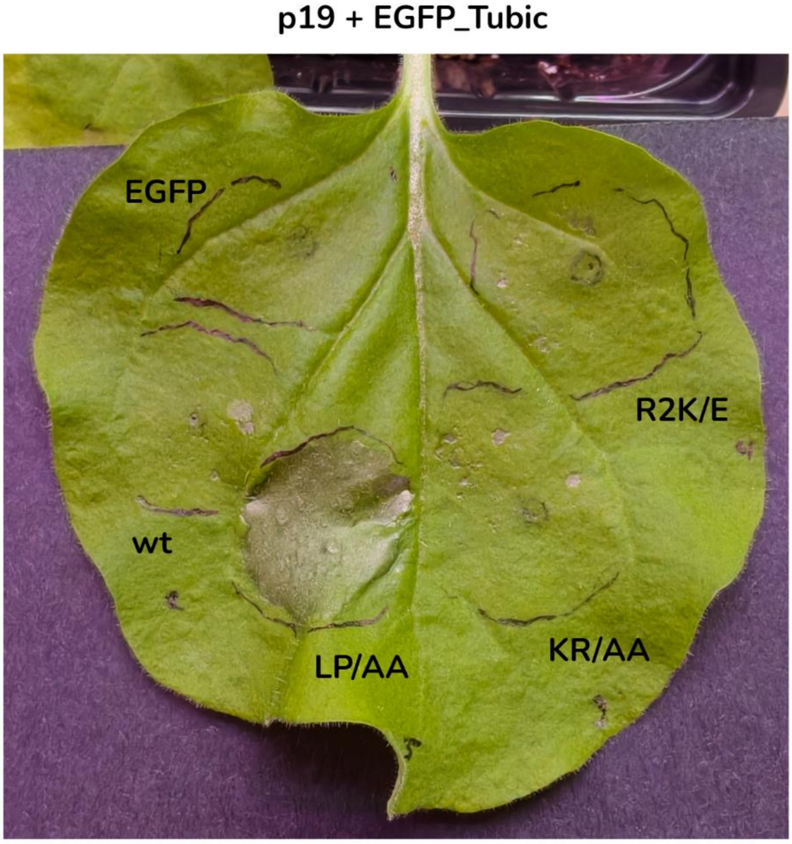

Supplementary Figure S2. Production of the F-box Tubic mutant LP/AA causes early necrotization in a *N. benthamiana* leaf, whereas leaf areas producing wild type Tubic (wt), the KR/AA, and R2K/E Tubic mutants preserve cell viability. An image was obtained at 3 dpi.

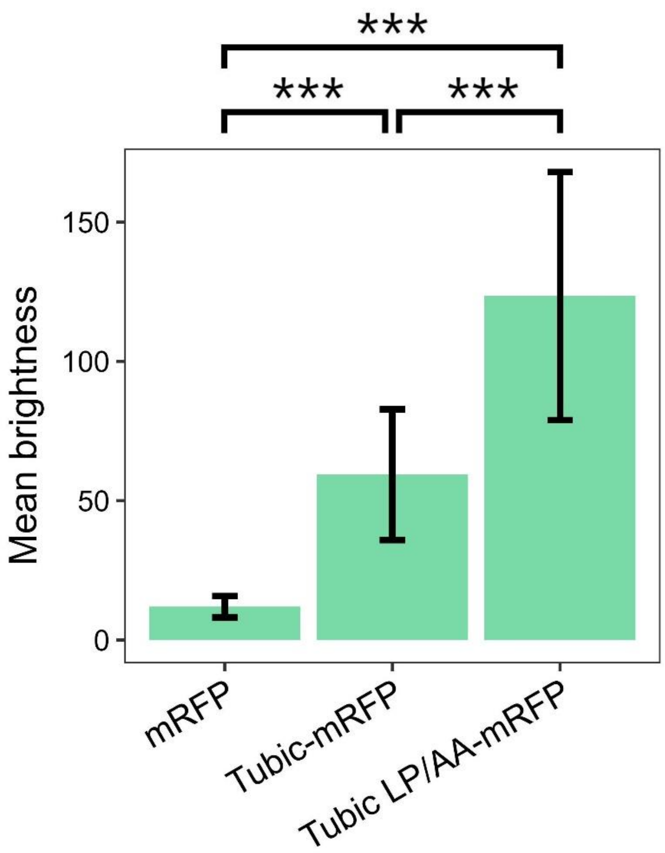

Supplementary Figure S3. Quantification of ROS generation (using H2DCFDA staining) in *N. benthamiana* leaves producing Tubic-mRFP, Tubic LP/AA-mRFP, or free mRFP at 2 dpi. Fluorescence microscopy images obtained with identical microscope settings were used for statistical analysis. The fluorescence signal of the nucleus of each cell in the image (13-23 samples for each group) was measured using the ImageJ Fiji program. Data are presented as means ± SD. Statistical significance was analyzed using a *t*-test with Benjamini-Hochberg correction, $p < 0.05$ was considered significant.

Supplementary Table S1.

List of the primers used in this study.

#	Primer name	Sequence
1	NtTubic_Nco_dir	CAA CCA TGG CCT CTT TCC GCA GTA TCG CTC G
2	Leader_Bam_rev	CAG GAT CCG TGC TTT GAA TGA CTA TAG GC
3	Fbox_Bam_rev	CAG GAT CCT TAA GAG AGA CCG GAA AG
4	NtTub_deltaN_Nco_dir	CAG CCA TGG CTA GTT GTT GGG CAA GCC TC
5	NtTubic_Bam_rev	GTG GAT CCT GTG CTT CAC ATG CCA ACT TTG TG
6	NtTub_Tubdom_Nco_dir	GAC CCA TGG TCT CTC TTA AGC AGC C
7	pLH_seq_dir	GAC CTC GAG AAT TCT CAA C
8	Tub_leader_Afl_rev	CTG CTT AAG GCT TTG AAT GAC TAT AGG
9	NtTubic_KR/AA_rev	GTG CCC AGA AAA TTT GAA GCC AGA GCT CCA ATA TAA GAG CTG CTT G
10	Tubic_K356E_dir	CCG TCG CTT CTG TCG AGA ATT TCC AGC TGA TTG C
11	Tubic_K392E_dir	GAC AAG ATT ATC TTG CAG TTT GGT GAG GTT GGC AAA GAT ATG
12	Tubic_R130E_Psi_rev	GGT AAG TTG TAT TAG CTT TGT CTT CTC TTA TAA AGC ATT GGA TGG TTC C
13	INF_Not_dir	CAC GCG GCC GCA CCA CGT GCA CCA CCT CGC AGC
14	INF_Sac_Xho_rev	GTG CTC GAG CTC ATA GCG ACG CAC ACG TAG ACG
15	NbTub1_qPCR_dir	AGT TCA TAA TTT ATG ATA CG
16	NbTub14_qPCR_dir	AGT TCA TAA TCT ATG ATA CC
17	NbTub_qPCR_rev	CAT GTG CAA TTT TGT AGC

Supplementary Table S2.

The exact domain boundaries of the Tubic derivatives constructed in this study. Amino acid numbering corresponds to wild type Tubic protein.

Name of the Tubic mutant	Starting from ….. terminating with
ΔN	^{51}SCWAS….KLACE^{426}
ΔF	^{2}SFRSI…..IVIQS^{50} ^{114}LKQPG…..KLACE^{426}
ΔNF (Tubby domain)	^{112}VSLKQ…..KLACE^{426}
NF (ΔTubby domain)	^{2}SFRSI…..PVSLK^{115}
N	^{2}SFRSI…..IVIQS^{50}

Supplementary Table S3.

Occurrence of predicted contacts between selected Tubic residues and the phosphate group of PA across computational models. A contact was defined as a distance of ≤4.0 Å between heavy atoms of the residue and atoms of the PA.

Residue	Chai-1	Boltz-2	IntelliFold	DynamicBind
Arg130	10/10	0/10	5/10	1/5
Lys356	10/10	10/10	8/10	0/5
Lys392	10/10	10/10	8/10	3/5

Supplementary Table S4.

Summary of confidence metrics for computational models of the Tubic–PA complex.

Method	Confidence metric	Number of models	Mean score	SD	Range
Chai-1	Aggregate score	10	0.227	0.009	0.212-0.240
Boltz-2	Confidence score	10	0.658	0.008	0.646-0.675
IntelliFold	Ranking score	10	0.457	0.036	0.392-0.497
DynamicBind	IDDT	5	0.358	0.037	0.305-0.397
